# An algorithm for automated ROI definition in water or epoxy‐filled NEMA NU‐2 image quality phantoms

**DOI:** 10.1120/jacmp.v17i1.5842

**Published:** 2016-01-08

**Authors:** Larry A. Pierce, Darrin W. Byrd, Brian F. Elston, Joel S. Karp, John J. Sunderland, Paul E. Kinahan

**Affiliations:** ^1^ Department of Radiology University of Washington Seattle WA; ^2^ Department of Radiology University of Pennsylvania Philadelphia PA; ^3^ Department of Radiology University of Iowa Iowa City IA USA

**Keywords:** NEMA NU‐2, segmentation, automation, quantitation

## Abstract

Drawing regions of interest (ROIs) in positron emission tomography/computed tomography (PET/CT) scans of the National Electrical Manufacturers Association (NEMA) NU‐2 Image Quality (IQ) phantom is a time‐consuming process that allows for interuser variability in the measurements. In order to reduce operator effort and allow batch processing of IQ phantom images, we propose a fast, robust, automated algorithm for performing IQ phantom sphere localization and analysis. The algorithm is easily altered to accommodate different configurations of the IQ phantom. The proposed algorithm uses information from both the PET and CT image volumes in order to overcome the challenges of detecting the smallest spheres in the PET volume. This algorithm has been released as an open‐source plug‐in to the Osirix medical image viewing software package. We test the algorithm under various noise conditions, positions within the scanner, air bubbles in the phantom spheres, and scanner misalignment conditions. The proposed algorithm shows runtimes between 3 and 4 min and has proven to be robust under all tested conditions, with expected sphere localization deviations of less than 0.2 mm and variations of PET ROI mean and maximum values on the order of 0.5% and 2%, respectively, over multiple PET acquisitions. We conclude that the proposed algorithm is stable when challenged with a variety of physical and imaging anomalies, and that the algorithm can be a valuable tool for those who use the NEMA NU‐2 IQ phantom for PET/CT scanner acceptance testing and QA/QC.

PACS number: 87.57.C‐

## I. INTRODUCTION

In positron emission tomography (PET), the resolution and contrast recovery properties of a PET scanner are often reported by using the Medical Imaging and Technology Alliance (MITA, a division of the National Electrical Manufacturers Association, NEMA) NU‐2 Image Quality (IQ) phantom protocol.^1^, ^2^, ^3^ The IQ phantom consists of a plastic body (an approximation of a human torso) filled with an aqueous solution of ${\;}^{18}F$ (typically using ^18^F‐flourodeoxyglucose (FDG), the most common PET radiotracer). Within the body are six tillable spheres with interior diameters ranging from 10 mm to 37 mm, arranged in a hexagonal pattern. The NEMA NU‐2 standard requires that the largest two spheres be filled with water (no activity from FDG) and the four smallest spheres be filled with FDG with an activity concentration ratio of 4:1 or 8:1 over the background activity. A 5 cm diameter cylindrical insert runs through the center of the phantom. This insert is generally packed with material to simulate lung attenuation coefficients. The phantom is then subjected to a positron emission tomography/computed tomography (PET/CT) scan.

**Figure 1:**
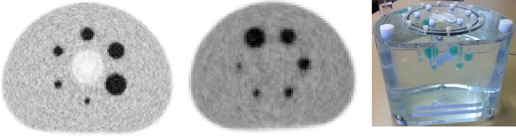
Two PET images (left and center) and a photo (right) of the NEMA NU‐2 IQ phantom. The PET image on the left includes the cold lung insert through the middle of the phantom, while the image in the center does not. Note that all spheres have activity above the background and are not in the same relative position between the two PET scans.

Due to its wide availability and modular construction, the NEMA NU‐2 IQ phantom has found uses in other settings, often with modifications, such as filling all six spheres with FDG^(4)^ or omitting the lung insert (Fig. 1). The IQ phantom has also been used for harmonization studies in which the phantom is filled with ${\;}^{68}\text{Ge}$ doped epoxy of carefully measured activity concentration.^5^, ^6^, ^7^, ^8^ This epoxy‐filled phantom is sent to multiple clinical sites in order to ensure quantitative agreement among a network of sites. The European Association of Nuclear Medicine (EANM) promotes the use of a modified IQ phantom in an “initiative to promote multicenter nuclear medicine and research”.^(4)^ Many clinical trials involving PET quantitation prefer that a site undergo tests with the IQ phantom in order to improve quantitative agreement, thus increasing the power of the trial. The IQ phantom has also been the model for a digital reference object for the evaluation of PET/CT imaging software.^(9)^

In any configuration, scans of the IQ phantom are then subjected to 2D or 3D region of interest (ROI) tests. In these tests, a user draws 2D circular or 3D spherical ROIs around each of the six spheres, as well as several ROIs within the background (body) of the phantom. Scanner and reconstruction‐specific properties are then computed from the measured ROI values.

While the NEMA NU‐2 standard specifies that “the diameters of the ROIs shall be as close as possible to the physical inner diameters of the spheres”,^(1)^ the ROIs are generally positioned manually, allowing for variability among different human readers, especially when the smallest spheres are difficult to identify within the PET image volume. Furthermore, manual placement of the ROIs is a time‐intensive task, often requiring ROIs to be drawn on multiple scans. And while the maximum voxel value can generally be determined by drawing a rough bounding box around each sphere, the mean value is highly dependent in how the ROI is drawn.

In order to free up the operator time needed for manual ROI placement, an automated method for generating ROIs for the IQ phantom is proposed. However, there are several challenges to creating such an algorithm.

Often, the smallest spheres are difficult to see in the PET image volume. This makes a sphere‐shaped matched filter or a thresholding algorithm poor candidates for finding the smallest spheres, as these are sensitive to PET image noise. While one can theoretically define ROIs for the smallest spheres according to the phantom specifications, in practice the physical positions of the spheres can be 5 mm or more from their theoretically prescribed positions. Indeed, every time an IQ phantom is disassembled and reassembled, it should be anticipated that the relative positions of the six spheres are altered. Furthermore, the spheres are not always in the same winding orientation (e.g., clockwise increasing diameter) or placement (e.g., small sphere on bottom vs. small sphere at left) as shown in Fig. 1. This inter‐ and intraphantom sphere position variability makes a fixed, universal, six‐sphere matched filter a poor candidate for an automated ROI placement algorithm.

Another challenge to automated ROI placement is the presence of air bubbles in the spheres. Air bubbles are regularly present in the sphere stems after filling and these often find their way up the stems and into the spheres. These bubbles have no FDG activity and very low attenuation — facts that can confound matched filter localization in both the PET and CT image volumes. Any automated algorithm for IQ phantom sphere localization should have the ability to detect air bubbles within the spheres and compensate accordingly.

Many methods have been used to perform the localization of IQ phantom spheres; however, the authors are unaware of any publications describing these algorithms or the results of their use. One method utilizes a user‐guided oversampling of the PET image volume to maximize activity concentrations. Unfortunately, pattern matching on an oversampled grid is extremely slow and restricts the sphere centers to that grid. Another method uses a user‐seeded, region‐growing edge detection, or other segmentation, on the interior of the CT spheres, and follows with a rigid registration from the CT to the PET images (or simply uses the DICOM‐defined registration). Since rigid registration algorithms can vary depending on the software package, some variability in the result would be expected from this technique. There may even be variability within a single PET/CT image set if this method is run multiple times. Furthermore, not all image viewing software packages have automated rigid registration built into them. In both cases, operator intervention is needed to guide the algorithm. The software from most major vendors has built‐in NEMA testing software that can report the mean ROI values, but these are generally not publicly available.

Despite the IQ phantom's widespread use as a standard tool for PET/CT scanner qualification, analyses of image sets are subject to analysis inefficiencies and a lack of standardized reporting. This work reports a fully automated analysis approach that addresses these limitations through a fully automated algorithmic approach that uses information from both the PET and CT images to overcome the challenges of localizing the smallest PET spheres. The software is currently implemented as an open‐source plug‐in to the Osirix DICOM viewing software package (Pixmeo, Bernex, Switzerland).

The proposed algorithm is easily altered for situations when the spheres are hot or cold, and is amenable to being used with the ${\;}^{68}\text{Ge}$ epoxy phantom. For descriptive purposes, we present the algorithm for finding six hot spheres and discuss modifications in the Discussions section. This algorithm was implemented and tested for accuracy and robustness to errors in positioning of the phantom and relative positions of the spheres. We also tested the algorithm for robustness against various noise conditions, and its ability to detect and compensate for air bubbles in the phantom spheres.

## II. MATERIALS AND METHODS

The NEMA NU‐2 IQ phantom contains six tillable spheres with specified interior diameters of 10, 13, 17, 22, 28, and 37 mm, which we will refer to as spheres 1 through 6, with sphere 6 being the largest sphere. The plastic walls of each sphere are 1 mm thick. The centers of these spheres in relation to the phantom body are specified according to the NEMA NU‐2 documentation.^1^, ^2^

The algorithm described here is designed for the NEMA NU‐2 IQ phantom modification in which all six spheres are filled with FDG above the background concentration (six hot spheres) and was implemented using MATLAB (MathWorks, Natick, MA). The PET and CT image volumes are loaded from the DICOM files. The Image Orientation Patient DICOM field (0020, 0037) is checked to assure image slices are orthogonal by verifying that each (x, y, z) value is one of {−1,0,+1}. If they are not, the image slices are deemed ‘nonorthogonal’, and the program reports this to the user and terminates.

**Figure 2:**
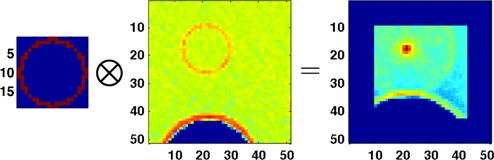
Illustration of matched filtering. A binary ‘matched filter template’ (left) is multiplied point wise across the image volume (center) to obtain the matched filter values (right). The matched filter value space is the same size as the image volume, while the template is smaller. Matched filter values are recorded at the center voxel of the template. Note that the edges of the matched filter value space are not populated due the template hitting the boundary of the image space.

We adopt the notation that a ‘matched filter’ consists of a binary ‘matched filter template’ that is point‐wise multiplied with the image volume to obtain a ‘matched filter value space’, as illustrated in Fig. 2. For each position within the image volume, the template is overlaid, and the sum or mean of point‐wise multiplication is taken to populate the matched value space.

### A. Overview of the algorithm

**Figure 3:**
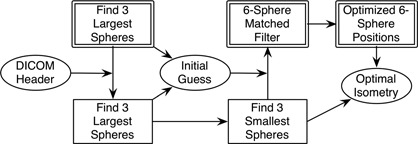
Workflow of the algorithm. Double‐outlined boxes indicate operations in the PET image volume, while single‐lined boxes indicate operations in the CT volume. Ellipses indicate isometry maps between the PET and CT image volumes, initially using the DICOM information and ending with the optimized map from PET to CT coordinates. The DICOM and optimized maps are compared to determine scanner misalignment. The optimal six‐sphere PET positions can be used to draw ROIs in the PET volumes. The bubble detection algorithm happens within each CT operation.

The proposed algorithm uses both the PET and CT image volumes of the IQ phantom as outlined in Fig. 3. The algorithm proceeds according to the steps outlined here:

1. Sphere‐shaped matched filters are used to estimate the centers of the three largest spheres within the PET image volume.
2. The DICOM header information is used to map the estimated centers of the largest spheres into the CT image volume.
3. The centers of the three largest spheres are accurately determined within the CT volume by using matched filters in a local search (defined by a bounding box) near the estimated position. A test is performed to check for air bubbles within the spheres. Any voxel categorized as ‘air’ is excluded from the matched filter.
4. The centers of the three largest spheres determined in Step 3 are used to determine which voxels are considered to be part of the plastic sphere shell for each of the largest three spheres, excluding those categorized as ‘air’. These voxel values are recorded.
5. The centers of the three smallest spheres are estimated within the CT volume by extrapolating the anticipated descending‐diameter hexagonal pattern of sphere centers from the centers of the three largest spheres.
6. The centers of the three smallest spheres are accurately determined by using matched filters in a local search (defined by a bounding box) near the estimated centers. The voxels values of the plastic spheres learned in Step 4 are used for the matched filter. A test for air bubbles in the spheres is performed and voxels categorized as ‘air’ are excluded from the matched filter.
7. An initial guess of the isometry from the CT to the PET image volume is created by fitting the centers of the three largest spheres in the CT and PET volumes.
8. A six‐sphere matched filter template is created within the PET volume by using the isometry estimated in Step 7 applied to the six CT sphere centers determined in Steps 3 and 6.
9. The matched filter template from Step 8 is perturbed with six degrees of freedom within the PET volume. The best‐fit isometry from the CT to the PET is computed from this matched filter perturbation and the centers of all six spheres are recorded within the PET volume.
10. The sphere centers determined in Step 9 are output to the user and can be used to draw 2D or 3D ROIs for the test being performed. In the Osirix implementation, these are used to automatically define the corresponding 3D ROIs, and a report of the ROI measurements is generated as a text file.

### B. Details of the algorithm

#### B.1 Estimation of the centers of the largest sphere center in PET

For the largest sphere, a spherical matched filter template for the PET image volume is created. This matched filter is binary and is generated assuming the sphere center is the exact center of a PET voxel. Voxels in the template are given a value of ‘1’ only if the center of the voxel lies within or on the theoretical sphere boundary, a method we refer to as the ‘Binary Center Inclusion Method’.

This filter template is then matched to the entire PET image volume, and the sum of the image volume voxels within the template is used to obtain a three‐dimensional set of matched filter values. The argmax voxel position (the voxel position that generates the maximum matched value within the matched value space) is chosen as the initial estimate of the largest sphere and is recorded as $C_{P,6,E}$ (center of PET sphere 6 estimate).

The voxels corresponding to this template at the argmax position are then set to zero, and the process is then repeated to estimate the center of the second‐largest sphere, $C_{P,5,E}$. (Setting the largest sphere voxels to zero prevents the next iteration from finding the largest sphere again). This is done once again to estimate the center of the third‐largest sphere, $C_{P,4,E}$.

#### B.2 Accurate localization of the three largest spheres in CT

The positional information from the CT and PET DICOM headers is used to create an isometry, $\Phi_{\text{DICOM}}$, from the CT into the PET image volumes. (This isometry is in millimeters, not image voxels).

The estimates of the three largest sphere centers in PET voxel coordinates from the Materials & Methods section B.1 above. ($C_{P,6,E}$, $C_{P,5,E}$, and $C_{P,4,E}$) are mapped to CT voxels coordinates by applying $\Phi^{-1}{\;}_{\text{DICOM}}$ to each point to obtain points $C_{C,6,E}$, $C_{C,5,E}$, and $C_{C,4,E}$, the subscript ‘C’ indicating these are points in the CT volume.

For each of the points $C_{C,6,E}$, $C_{C,5,E}$, and $C_{C,4,E}$, a three‐dimensional bounding box is then drawn around the point in the CT volume. The bounding box is defined around each point so that the number of voxels in each dimension is the minimum number needed to include two sphere diameters. This bounding box is then used to extract a subvolume from the CT image volume.

For each of the largest three spheres and the corresponding CT subvolume, a matched filter template is defined as a hollow spherical shell in CT voxels. Each binary filter template assumes the sphere center is the center of a CT voxel and is generated with the NEMA NU‐2–defined inner diameter and a sphere shell thickness of 1 mm using the Binary Center Inclusion Method described in the Materials & Methods section B.1.

For each CT subvolume, the voxel values are analyzed and voxels are categorized as ‘air’, using the methods described in Materials & Methods section B.4. The filter template is then matched against the subvolume by computing the mean value of nonair voxels in the template. The matched template corresponding to the argmax of these values is then used to identify nonair voxels as ‘plastic sphere wall’. (If multiple maxima are found, the first one found is chosen and the user is warned.) The mean of all voxels labeled as ‘plastic sphere wall’ is computed and recorded as $\mu_{\text{PSW}}$.

A subset of the CT subvolume‐matched filter value space is chosen as the largest box around the maximum such that the X, Y, and Z profiles through the maximum matched value have strictly negative curvature (concave down). A 3D quadratic is then fitted to this subset and the analytically computed vertex of that quadratic is defined to be the accurate center of the sphere. We call these points $C_{C,6,A}$, $C_{C,5A}$, and $C_{C,4,A}$, the subscript ‘A’ indicating these are the ‘accurate’ CT sphere center points. (Note that while the X, Y, and Z profiles in this subset all have negative curvature, the curvature in other regions of the subset may not be negative.)

#### B.3 Accurate localization of the three smallest spheres in CT

Estimates of the centers of the three smallest sphere centers are extrapolated from the points $C_{C,6,A}$, $C_{C,5,A}$, and $C_{C,4,A}$, according to the anticipated descending‐diameter hexagonal pattern. This is done by first computing the center of the hexagon within the CT volume as $C_{C,H}=C_{C,4,A}+C_{C,6,A},C_{C,5,A}$. The estimates of the three smallest sphere centers are computed by reflecting the centers of the three largest spheres through the hexagon center: $C_{C,i,E}=2\times C_{C,H}-C_{C,i+3,A}$ for i∈{1,2,3}.

A three‐dimensional bounding box is drawn within the CT volume around each of the points $C_{C,3,E},C_{C,2,E}$, and $C_{C,1,E}$ such that the number of voxels in each dimension is the minimum number needed to include three sphere diameters. If the CT voxels are greater than 1 mm in any dimension, the CT subvolume is up‐sampled so that the new CT voxel size is the minimum needed to make the subvolume voxels less than 1 mm in that dimension. This is an integer up‐sampling with no interpolation. (This is needed due to the sphere walls being nearly 1 mm thick.)

The up‐sampled subvolume voxel values are then analyzed and voxels are identified as ‘air’, using the methods described in Materials & Methods section B.4. The mean of the voxels labeled as ‘plastic sphere wall’, $\mu_{\text{PSW}}$ from Materials & Methods section B.2, is then used to rescale the up‐sampled image subvolume values as the absolute difference from the mean value of the plastic sphere voxels:

(1) $$V_{new}=-\left|V_{old}-\mu_{PSW}\right|$$

where $V_{\text{new}}$ and $V_{\text{old}}$ represent the new and original voxels values in the up‐sampled CT subvolume.

A binary matched filter template is then created for the three smallest spheres according to a hollow sphere with NEMA NU‐2–defined inner sphere diameter and plastic shell thickness of 1 mm, centered at the center of a CT voxel. This filter template is then matched to the $V_{\text{new}}$ voxel values, computing the mean values over nonair voxels within the template for each position. If multiple maxima are found in the matched values, the first position found is used as the maximum and a warning is displayed to the user.

A subset of the $V_{\text{new}}$ up‐sampled subvolume matched values is then determined as the maximal subset, such that X, Y, and Z profiles through the maximum matched value have negative curvature, as in Materials & Methods section B.2. A 3D quadratic fit is done to this subset and the analytically computed vertex of the quadratic fit is then taken to be the accurate sphere center. This gives the points in CT coordinates $C_{C,3,A},C_{C,2,A}$, and $C_{C,1,A}$.

#### B.4 Determination of air bubbles in the CT volume

In Materials & Methods B.2, B.3, bounding boxes are drawn around each of the spheres in the CT volume and subvolumes are analyzed to determine if any air bubbles are present.

For each CT subvolume examined, the voxel values of the subvolume are histogrammed into 10 bins of equal width from the minimum to the maximum subvolume voxel values. If the mode of the histogram is in the upper half of the histogram (bins 6−10), then it is determined that voxels representing air are present within the CT subvolume. (The subvolumes are sufficiently large that the majority of voxels should have a value corresponding to water). In this case, each voxel with a value that is 2 standard deviations (2 SD) or more below the subvolume voxel mean is labeled as ‘air’.

In B.2, B.3 above, the mean of nonair voxels from the matched filter template is used. However, if air is not detected within a subvolume, the sum is used instead of the mean to speed computation time.

#### B.5 Initial estimation of the isometry from CT to PET

An initial estimate of the isometry from CT to PET coordinates, $\Phi_{E}$, is computed by mapping the centers of the largest three spheres in CT space near to the estimates of those centers in PET coordinates ($C_{P,6,E},C_{P,5,E}$, and $C_{P,4,E}$, as computed in section B.1 above). We define $\Phi_{E}$ as the isometry that satisfies the three conditions:

1. The center of CT sphere 6 is mapped onto the initial guess of the center of PET sphere 6: $\Phi_{E}(C_{C,6,A})=C_{P,6,E}$;
2. The line in CT coordinates through the points $C_{C,6,A}$ and $C_{C,5,A}$ is mapped onto the line in PET coordinates through the points $C_{P,6,E}$ and $C_{P,5,E}$; and
3. The plane in CT coordinates defined by the points $C_{C,6,A}$, $C_{C,5,A}$, and $C_{C,4,A}$ is mapped onto the plane in PET coordinates defined by the points $C_{P,6,E}$, $C_{P,5,E}$, and $C_{P,4,E}$.

Due to symmetries, there are two transforms that satisfy condition 2 and two more for condition 3. At each step, the transform that minimizes the Euclidean distances $∥\Phi_{E}(C_{C,i,A})-C_{P,i,E}∥$ is chosen. If these conditions cannot be met for any reason (e.g., the three points are collinear), the user is notified and the algorithm terminates.

#### B.6 Computation of the best fit isometry from CT to PET

The six CT sphere centers, $C_{C,i,A})$ are then mapped via $\Phi_{E}$ (computed in section B.5 above) into PET voxel coordinates. A six‐sphere matched filter template for the PET volume is generated by creating a filled binary sphere of the appropriate diameter around each of the points $\Phi_{E}$($C_{C,i,A}$) and using the Binary Center Inclusion Method described in section B.1 above. (Note that the center of each sphere may not be the center of a voxel.)

The isometry, $\Phi_{E}$, is then perturbed in six degrees of freedom $\left(\theta_{x},\theta_{y},\theta_{z},\Delta_{x},\Delta_{y},\Delta_{z}\right)$, where ($\theta_{x},\theta_{y},\theta_{z}$) denote rotations about the x‐, y‐, and z‐axes (not Euler angles) and ($\Delta_{x},\Delta_{y},\Delta_{z}$) denote translations in the X, Y, and Z coordinates. For rotations, we use the convention that the centroid of the points $\Phi_{E}$($C_{C,i,A}$) is the origin.

The $\left(\theta_{x},\theta_{y},\theta_{z}\right)$ perturbation increments are defined such that one increment of $\theta_{x}$ (similarly $\theta_{y}$ and $\theta_{z}$) results in the theoretical sphere centers being rotated about their centroid by 1 mm up to a range of ±3 mm. The $\left(\Delta_{x},\Delta_{y},\Delta_{z}\right)$ translation perturbation increments are 1 mm up to a range of ±3 mm. For each $\left(\theta_{x},\theta_{y},\theta_{z},\Delta_{x},\Delta_{y},\Delta_{z}\right)$ perturbation to $\Phi_{E}$, the points $C_{C,i,A}$ are mapped into PET coordinates and a new six‐sphere matched filter template is generated. This template is matched against the PET volume voxels and the sum of voxels within the template is recorded in the six‐dimensional matched value space.

A six‐dimensional quadratic is then fitted to matched filter value space, and the analytically computed vertex of that quadratic is used to define $\Phi_{O}$, the ‘optimal isometry’ from CT to PET voxel coordinates.

If the maximum of the matched values occurs at the boundary of the matched value space in any dimension, a warning is reported to the user. Also, if the vertex of the computed quadratic is not within two indices of the maximum within the matched value space, a warning is reported to the user. These warnings alert the user that the quadratic fit may not be accurate, as quadratic fitting works best if the vertex is in the interior of the matched value space.

The algorithm then reports the accurately positioned PET sphere centers, $C_{P,i,A}$, to the user as $C_{P,i,A}=\Phi_{O}(C_{C,i,A})$ for i∈{1,…,6}. These points can then be used by the user to define 2D or 3D ROIs around the sphere center to extract metrics for the test being performed.

#### B.7 Algorithm modifications for the epoxy phantom

For the ${\;}^{68}\text{Ge}$ epoxy phantom described in the Introduction and the Results section C.2 below, Steps 3−6 were performed manually on a single high‐resolution CT scan of the ${\;}^{68}\text{Ge}$ epoxy phantom. This scan was performed on a GE Discovery STE (GE Healthcare, Waukesha, WI) at 140 kVp and 300 mA. The reconstructed image volume was 512×512×120 voxels of size $0.43\times 0.43\times 0.625\;{\text{mm}}^{3}$.

The bounding box for each CT subvolume was defined manually. The matched filter templates were also manually manipulated by the user to exclude voids present in the epoxy and the stems attached to the plastic spheres. This was done due to the fact that the epoxy and plastic sphere walls have similar attenuation coefficients, making automated CT sphere localization difficult for the epoxy phantom.

These steps were performed only once, since the relative positions of the spheres do not change over time on the epoxy phantom. Note that Step 1 (localizing the three largest PET spheres) is performed as normal, but Step 2 (mapping the PET spheres into the CT volume using the DICOM header information) is not necessary for the epoxy phantom scans.

### C. Testing the algorithm

#### C.1 Testing the CT sphere localization algorithm

An IQ phantom was filled with water (not FDG) and a series of CT scans were taken on a GE DSTE scanner set to 120 kVp and 60 mA with pixel sizes of $1.37\times 1.37\;{\text{mm}}^{2}$ and a slice thickness of 2.5 mm, similar to that used clinically for a PET/CT scan.

**Figure 4:**
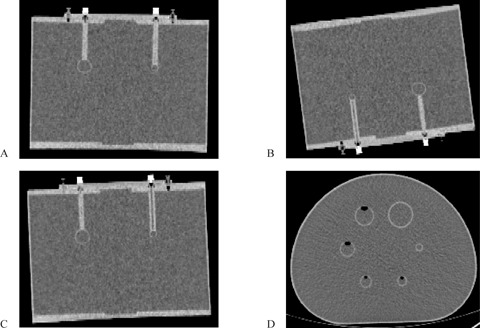
Coronal CT images of the IQ phantom in positions A (top‐left), B (top‐right), and C (bottom‐left), together with an axial view of position C to show the bubbles in spheres 2–5.

The phantom was placed on the scanner bed and 50 consecutive CT scans were taken. The phantom was then moved on the bed (rotated roughly 150°) and another series of 50 CT scans were taken in the new position. The phantom was then opened and small air bubbles were injected into spheres 5, 4, 3, and 2 (all but the largest and smallest spheres). The phantom was then scanned another fifty times in yet a third position on the scanner bed. We refer to each series of CT scans as positions A, B, and C, with C being the scans with bubbles. Images of the three scan positions and bubbles are shown in Fig. 4.

For each of the three phantom positions, a single scan was viewed, and a user selected a point near the center of spheres 6, 5, and 4. This step is only necessary for testing the algorithm; the user never performs this task. This is because the points $\Phi^{-1}{\;}_{\text{DICOM}}$($C_{P,i,E}$) computed in Materials & Methods section B.2 are not available due to the lack of a corresponding PET scan. These points were then input into the algorithm and the accurate localization of all six spheres was performed, as described in B.2, B.3 above, for each of the 50 scans.

The standard deviation of the computed sphere centers for each position on the bed is used to quantify the stability of the algorithm. For each of the three positions on the bed, the mean and standard deviation of the 50 (x,y,z) sphere center positions, denoted $\mu_{X,i}$ and $\sigma_{X,i}(X\in \{A,B,C\},i\in \{1,\ldots ,6\})$ respectively, was computed in millimeters for each sphere. These standard deviations are reported in Table 1.

We then use the distances from mean sphere center positions to evaluate the positional stability for different bed position scans. If the distances between sphere centers remain constant between scans, then we infer that the sphere centers can be obtained by isometry from one phantom position on the bed to another and, therefore, the PET sphere localization will not be affected.

**Table 1:** The SDs (in mm) of CT sphere localization in X, Y, and Z. The left column indicates the sphere number (S1–S6), and the columns denote the three different positions of the phantom, A, B, and C. The last column, $C_{\text{NoBubbleDetection}}$ is used to denote the algorithm run on C without using the air detection algorithm to compensate for bubbles.

	*A*			*B*			*C*			$C_{\text{NoBubbleDetection}}$		
	$\sigma_{x}$	$\sigma_{y}$	$\sigma_{z}$	$\sigma_{x}$	$\sigma_{y}$	$\sigma_{z}$	$\sigma_{x}$	$\sigma_{y}$	$\sigma_{z}$	$\sigma_{x}$	$\sigma_{y}$	$\sigma_{z}$
S1	0.14	0.17	0.41	0.11	0.18	0.15	0.11	0.18	0.53	0.13	0.18	0.44
S2	0.10	0.15	0.08	0.10	0.15	0.11	0.10	0.13	0.21	0.13	0.16	0.15
S3	0.09	0.16	0.05	0.06	0.16	0.06	0.05	0.14	0.05	0.06	0.15	0.07
S4	0.11	0.13	0.22	0.06	0.17	0.10	0.06	0.13	0.17	0.11	0.13	0.09
S5	0.05	0.15	0.58	0.05	0.15	0.43	0.05	0.13	0.10	0.29	0.14	0.17
S6	0.04	0.15	0.49	0.03	0.16	0.55	0.03	0.14	0.43	0.03	0.14	0.43

This is accomplished by computing the distance between the mean sphere centers, $D_{X,i,j}=∥\mu_{X,i}-\mu_{Y,j}∥$, for all pairs i and j (15 measurements for each position: $D_{X,1,2},D_{X,1,3}\cdots D_{X,5,6}$) and all positions X∈{A,B,C}. For two positions of the phantom, X≠Y (three combinations), we compute the measurement discrepancy of the distance between sphere i and j as $\Delta_{X,Y;i,j}=|D_{X;i,j}-D_{Y;i,j}|$ in order to quantify the difference in relative sphere positions between different positions of the phantom. The mean and maximum values of $\Delta_{X,Y;i,j}$ are computed and the $\Delta_{X,Y;i,j}$ are also presented in a histogram of all measurement discrepancies.

#### C.2 Testing PET sphere localization using dynamic PET scans

Three dynamic PET scans were taken of the epoxy phantom on a GE Discovery STE PET/CT scanner, each in a different position on the scanner bed. The first dynamic scan consisted of 50 time frames with 10 min per time frame for a total scan time of 500 min. The second dynamic scan consisted of 50 frames with 5 min per frame (250 min total scan time). The third dynamic scan consisted of 50 frames at 2.5 min per frame (125 min total scan time). The 5 min scans were performed two months after the 2.5 and the 10 min scans, resulting in lower activity concentrations for those scans.

Each dynamic scan was reconstructed using OSEM with 4 iterations and 28 subsets with Gaussian postfiltering kernels of 6 and 12 mm full width at half maximum (FWHM). Thus, we obtained a total of six dynamic image volumes. Each volume consisted of 256×256×47 voxels of size $2.73\times 2.73\times 3.27\;{\text{mm}}^{3}$.

For each combination of 50 frames and six reconstructions, the PET sphere center estimates $C_{P,6,E}$, $C_{P,5,E}$, and $C_{P,4,E}$ were computed as in section B.1 above. The optimal isometry, $\Phi_{O}$, was computed from these estimates and the predefined CT sphere centers for the epoxy phantom described in the Materials & Methods section A. We then recorded the (x,y,z) centers in millimeters of the six spheres in the PET volume, $C_{P,i,A}=\Phi (C_{C,i,A})$.

For each position of the phantom, we computed the standard deviation (in millimeters) of the sphere centers in the X, Y, and Z directions. We also recorded the mean and maximum voxel value within the six‐sphere ROI for each time frame. The standard deviation and coefficient of variation were then computed for the set of mean ROI values and for the set of max ROI values.

#### C.3 Testing the air bubble detection algorithm

**Figure 5:**
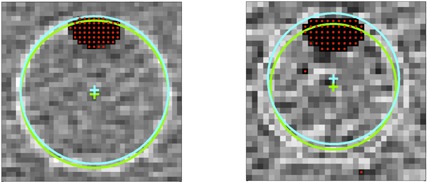
CT images of spheres 3 and 4 with bubbles. The voxels with a red dot are tagged as ‘air’ in the algorithm. The blue circle shows the detected sphere interior boundary without using the bubble exclusion method; the blue cross is the center of that circle. The green cross and circle show the detected boundary determined by excluding the air voxels. Note that the radii of the circles are not equal, as the sphere center occurs in different slices.

In order to evaluate the air bubble detection algorithm and its impact on CT sphere localization, we examined a single CT scan with visible air bubbles in spheres 3 and 4 (not one of the realizations from the 50 scans in position C from section C.1 above). The CT voxel sizes for this scan were $0.70\times 0.70\times 2.5\;{\text{mm}}^{3}$. The entire algorithm was run normally on the PET, and CT image volumes and the CT sphere centers were recorded. An image of spheres 3 and 4 for this CT scan are shown in Fig. 5.

The algorithm was then run again, but with the difference that no voxels were categorized as ‘air’. The difference (in mm) in the computed position of each CT sphere was then recorded. The centers and corresponding sphere boundaries computed with and without air detection are shown in Fig. 5.

We also recomputed each sphere localization from section C.1 above for position C (50 scans with air injected into spheres 2, 3, 4, 5) without using the air detection algorithm. The Euclidean distance between the CT sphere centers with and without air detection and compensation was computed and the maximum and mean over all 50 Euclidean distances were recorded.

#### C.4 Using the algorithm to detect scanner misalignment

**Figure 6:**
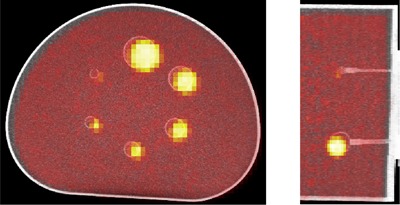
Axial and coronal PET/CT fusion images of the IQ phantom from the scanner with misalignment.

The algorithm was run on a PET/CT scan of the IQ phantom from a scanner known to have misalignment between the PET and CT image volumes. This scan is shown in Fig. 6. The isometry defined by the DICOM header information, $\Phi_{\text{DICOM}}$, was compared to the isometry determined by the algorithm $\Phi_{O}$ by computing the difference between mapped CT sphere centers, $D_{i}=\Phi_{O}(C_{C,i,A})-\Phi_{\text{DICOM}}(C_{C,i,A})$. We compute the norms, $∥D_{i}∥$, as well as the angular distribution of the $D_{i}$ as $\theta_{D}$, the maximum angle between the six $D_{i}$ vectors.

## III. RESULTS

### A. CT sphere localization results

Table 1 shows the standard deviations of the CT sphere localization algorithm over 50 scans for each of three positions of the phantom. The maximum standard deviation of the sphere localizations was 0.55 mm, with most results being under 0.20 mm.

**Figure 7:**
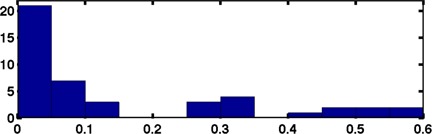
Histogram of the discrepancies of distances between spheres, $\Delta_{X,Y;i,j}$, for the mean sphere centers of positions X,Y∈{A,B,C} and i,j∈{1…6} (a total of 15 sphere‐pairs×3 position‐pairs for n=45 measurement discrepancies). The histogram bin width is 0.05 mm with 21 occurrences in the first bin. The mean discrepancy is 0.15 mm, and the max is 0.59 mm.

A histogram of the $\Delta_{X,Y;i,j}=|D_{X;i,j}-D_{Y;i,j}|$ is given in Fig. 7. The mean value for $\Delta_{X,Y;i,j}$ was 0.15 mm; the maximum was a 0.59 mm discrepancy between the distances from sphere 1 to sphere 4 between CT series A and B.

### B. Dynamic PET scan sphere localization results

Table 2 shows the results of the standard deviations of the (X,Y,Z) positions of the PET sphere centers $C_{P,i,A}$ for scan A (which is the most aligned with the scanner axis) and 6 mm postreconstruction smoothing. The results from scan positions B and C exhibited standard deviations comparable to those from scan A. For all scans, the 12 mm postreconstruction smoothing scans, the standard deviations were generally lower. For scan A, the standard deviation in millimeters was 22.24% lower for the 2.5 min scan, 17.02% lower for the 5 min scan, and 16.37% lower for the 10 min scan. Table 3 shows the results of the maximum and mean voxel values within the corresponding ROI.

**Table 2:** SDs (in mm) of sphere center position estimates for 50 ROI optimizations with the 6 mm post‐reconstruction smoothing from scan A.

		*10 mm Sphere 1*	*13 mm Sphere 2*	*17 mm Sphere 3*	*22 mm Sphere 4*	*28 mm Sphere 5*	*37 mm Sphere 6*
2.5 min	X	0.42	0.42	0.19	0.08	0.08	0.18
	Y	0.15	0.41	0.55	0.41	0.16	0.11
	Z	0.18	0.25	0.20	0.10	0.06	0.06
5 min	X	0.16	0.17	0.07	0.06	0.06	0.07
	Y	0.09	0.14	0.19	0.14	0.09	0.11
	Z	0.12	0.16	0.14	0.09	0.06	0.06
10 min	X	0.11	0.11	0.05	0.05	0.05	0.05
	Y	0.11	0.17	0.21	0.18	0.11	0.10
	Z	0.13	0.18	0.15	0.07	0.05	0.04

**Table 3:** Means, SDs, and coefficients of variation (σ/μ) for the max and mean ROI voxel values for 50 realizations each of the six reconstruction protocols. Note that the 5 min scans were performed two months after the other scans, resulting in lower activity concentrations.

		*ROI Max*			*ROI Mean*		
		*Mean*	*STD*	*COV*	*Mean*	*STD*	*COV*
2.5 min	6 mm	41,400	785.54	1.90%	25,933	110.98	0.43%
	12 mm	37,419	482.08	1.29%	22,857	87.66	0.38%
5 min	6 mm	30,909	706.18	2.28%	19,977	59.48	0.30%
	12 mm	28,572	373.50	1.31%	17,552	57.02	0.32%
10 min	6 mm	39,597	525.55	1.33%	26,350	54.69	0.21%
	12 mm	37,467	344.83	0.92%	23,167	46.29	0.20%

### C. Air bubble detection results

Figure 5 shows the results from running the algorithm with and without the bubble detection for spheres 3 and 4. The center of sphere 4 was shifted up 0.62 mm, right 0.01 mm, and axially 0.48 mm without bubble detection. The center of sphere 3 was shifted up 1.00 mm, left 0.01 mm, axially 0.68 mm.

The mean Euclidean distance between 50 sphere center localizations from CT series C with and without the air detection and compensation algorithm are 0.20 mm, 2.11 mm, 0.89 mm, 1.98 mm, 1.35 mm, and 0.00 for spheres 1–6, respectively. The maximum Euclidean distances were 0.39 mm, 2.47 mm, 1.04 mm, 2.19 mm, 1.77 mm, and 0.00 mm.

### D. Scanner misalignment results

Table 4 shows the differences between the isometries $D_{i}=\Phi_{O}(C_{C,i,A})-\Phi_{\text{DICOM}}(C_{C,i,A})$) and their norms. The maximum angle between difference vectors was 9.4°.

**Table 4:** Table of (X,Y,Z) differences (in mm) between CT sphere centers as defined in the DICOM header vs. our algorithm for the misaligned scanner: $D_{i}=\Phi_{O}(C_{C,i,A})-\Phi_{\text{DICOM}}(C_{C,i,A})$.

		*Sphere Number*					
		*1*	*2*	*3*	*4*	*5*	*6*
$D_{i}$	X	8.6	7.9	7.4	7.8	8.5	8.9
	Y	4.8	4.8	5.4	6.0	6.1	5.5
	Z	4.8	4.6	6.0	4.5	4.9	5.2
$∥D_{i}∥$		11.0	10.3	11.0	10.8	11.0	11.7

## IV. DISCUSSION

### A. Stability of the algorithm

The results of Table 1 show that we expect the CT sphere to differ less than 0.2 mm in X and Y, and less than 0.6 mm in the Z direction. The histogram in Fig. 7 shows that the relative positions of the spheres remain relatively isometric with respect to each other over multiple realizations and positions within the scanner, with most discrepancies being under 0.15 mm. Thus, we conclude that the CT sphere localization algorithm is robust against different orientations of the phantom within the scanner, as well as imaging noise.

The stability of the PET sphere center localization algorithm is evidenced by Table 2, which shows that for the noisiest dynamic scan (2.5 min with 6 mm smoothing), the estimated sphere centers generally vary less than one‐half of a millimeter. The largest variance was 0.55 mm for the 17 mm sphere. These image volumes had voxels of size $2.73\times 2.73\times 3.27\;{\text{mm}}^{3}$, thus we expect the center of each sphere to vary less than one‐fifth of a voxel even under noisy PET imaging conditions.

Table 3 shows that the max voxel values within the 50 PET ROIs have a coefficient of variation generally less than 2% (with the single exception of 2.3% for the 5 min 6 mm scan). The mean ROI voxel value had a coefficient of variation less than 0.5% for each of the six dynamic scans. Since the maximum voxel value can be found by using a loose bounding box around the spheres, we expect that these values will be the same under most algorithms. However, the ROI‐dependent calculation of the mean is stable, with the largest COV of 0.43% for the 2.5 min scan and 6 mm postsmoothing. We conclude that the PET localization algorithm is stable in the presence of imaging noise.

The stability of the localizations of CT and PET sphere centers directly translates into stability of ROI computations that depend on those sphere centers. Furthermore, we have shown that both the PET and CT localization methods work, even if the phantom is not aligned with the scanner axis. Thus careful placement of the phantom is not necessary for use with this method. Although we were not able to test the algorithm on a large set of PET/CT scans, we have demonstrated that each part of the algorithm is stable and accurate and, therefore, conclude that the overall algorithm will be stable and accurate. This is evidenced by the few PET/CT scans that we were able to obtain for testing. Indeed, the only missing link in our testing procedures is the initial estimation of the three largest CT sphere centers from the initial PET sphere estimates. However, the detection of the largest three spheres within the PET volume should be a trivial task with the matched filters used, and we would not expect the DICOM headers to exhibit misalignments greater than those seen in the Materials & Methods section C.4. The algorithm's ability to correctly localize the misaligned scan from section C.4 means that the algorithm is able to accurately localize CT centers, even in the most challenging situations.

Using a quadratic fit to the matched filter value space allows for subvoxel localization of sphere centers in a fast, analytic way. The use of quadratic fitting also helps the proposed algorithm to remain stable when presented with PET and CT imaging noise.

The algorithm is further stabilized by the statistical decomposition of the CT values into ‘water’, ‘air’, and ‘plastic sphere wall’ voxels (Materials & Methods B.2, B.4). This allows the algorithm to learn from the easy‐to‐find largest spheres to help localize the hard‐to‐find smallest spheres. The voxels corresponding to the smaller sphere walls are generally more subject to imaging noise simply because there are fewer voxels that represent them. Matching the values to the known mean values of ‘plastic sphere wall’ by the formula $V_{\text{new}}=-|V_{\text{old}}-\mu_{\text{PSW}}|$ allows us to create a matched filter for those spheres which does not simply find the maximum voxel intensity (which would be highly subject to noise when considering the small number of voxels that make up the plastic sphere). Rather, we are matching the voxel values against the expected intensity of the sphere walls. This makes the matching for the smaller spheres less likely to be affected by imaging noise.

Air bubbles within the spheres can bias the positioning of the CT sphere positions, as evidenced by the results of Results section 3.3 and Fig. 5 that show the difference in CT sphere localization with and without the bubble detection. The mean differences in CT sphere localization with and without air detection was large for spheres 2–5 (those with injected air), with a maximum up to 2.47 mm. Note that the air detection algorithm did not detect air in any of the images for sphere 6, thus the localizations for sphere 6 were exactly the same for all 50 scans.

The isometry histogram from Results section A shows that the CT localization algorithm is capable of retaining relative sphere distances, even when faced with bubbles in the spheres. The biases seen between localizations of spheres from CT series C with and without bubble compensation show that the isometries are broken when air bubble detection and compensation are not performed. We conclude that the air detection and compensation algorithm is stable and is a necessary part of the algorithm.

Our decision to use 2 SDs below the subvolume mean was based on the expectation that the majority of voxels in the subvolume would be water and that the only other materials in the subvolume would be air and plastic. Based on this expectation, 2 SDs were chosen as the cutoff. To ensure that the mean voxel value remained near that of water for the smaller subvolumes, we made the decision to expand the subvolumes to three sphere diameters for those smaller spheres. We believe that the decision to use 2 SDs below the subvolume mean is conservative, and can lead to nonair voxels within the sphere to be labeled as air, as evidenced by the two stray red dots in Fig. 5(b). Since these are likely due to imaging noise, we expect the miscategorized voxels to be randomly distributed and, therefore, not have a large effect on the position of the matched filter maximum or fitted quadratic.

Finally, it is important to note that the analytical nature of this algorithm makes it deterministic in that it will report the same values every time that it is run on the same set of PET/CT volumes.

### B. Alignment testing and algorithm modifications

The results from the Results section 4 above show that the norms of the $D_{i}$ indicate significant difference between the isometry determined by the algorithm and the isometry defined in the DICOM header. All differences in mapped CT sphere centers were greater than 10 mm, and the vectors in Table 4 agree with the visual differences visible in Fig. 6. The small angular distribution, $\theta_{D}=9.4^{\circ }$, of the difference vectors indicate that differences are all roughly in the same direction. Together, these give quantitative evidence that a misalignment is present between the PET and CT scans and therefore the scanner itself.

Thus, the algorithm can be used to warn a user of misalignment in the scanner, as any misalignments should propagate into the DICOM header information. It is for this reason that we chose not to use the DICOM header information to define the isometry between the CT and PET image volumes. However, the point at which the algorithm and DICOM header disagreement becomes significant has not yet been studied. Large values for the norms $∥D_{i}∥$ without tight angular distribution $\theta_{D}$ could indicate scaling issues rather than misalignment. For this reason, our algorithm only reports the values of the differences and the user must interpret those values to assess any misalignment.

The proposed algorithm is easily adaptable to situations where the largest spheres are cold by simply altering the corresponding matched filter template. This can be accomplished by subtracting the mean of the subvolume and using the negative of the matched filter template that was presented. Indeed, any configuration of hot and cold spheres can be entered into the template. The only information that the algorithm uses is that the spheres are in descending diameter in a roughly hexagonal array. Thus nonstandard sphere sizes (or even orientations) could be entered into the algorithm's initial conditions.

However, the sizes of the CT subvolume localization searches and the choice to only up‐sample the CT subvolumes around the three smallest spheres were made to optimize the algorithm for the standard NEMA NU‐2–specified sphere diameters. The size of the CT subvolume searches ensures that the subvolumes do not contain neighboring spheres. Up‐sampling the CT subvolumes around the three smallest spheres was found to be necessary in order to get filter templates that contained enough voxels to have numerical significance. For the larger spheres, the templates contain enough voxels that up‐sampling was found to be unnecessary. If nonstandard spheres were to be used, these conditions would likely need to be modified.

Other modifications that could be incorporated into the algorithm include the ability to read images with nonorthogonal image planes, the addition of voxel weights in the PET filter templates to reflect air within PET voxels, and the ability to automatically draw background ROIs within the phantom body. Another possibility is to draw 2D ROIs within the image once the sphere centers have been found.

Finally, we mention that this algorithm is particularly well‐suited to be used with the ${\;}^{68}\text{Ge}$ epoxy phantom,^6^, ^7^ since the initial steps of localizing the CT sphere centers only needs to be performed once for each phantom. Furthermore, since the orientation of the spheres does not change between successive scans, background ROIs can be predefined for the epoxy phantom. The epoxy phantom is sometimes scanned hundreds of times at each site, making ROI analysis extremely time‐intensive. Use of the proposed algorithm for batch processing of the epoxy phantom will greatly reduce the processing resources needed for that analysis.

### C. Study limitations

One limitation of this work is that we do not compare our algorithm to existing methods for ROI placement within the IQ phantom. In fact, the authors are unaware of any widely available automated algorithm for performing IQ phantom sphere localization. Nor are we aware of any testing that has been performed as to the variability of other techniques mentioned in the introduction.

Another limitation of this study is that, although we have shown the proposed algorithm to have low variability, we have not shown its accuracy. This is, of course, due to our lack of knowledge of the true sphere center positions. We can only say here that visual inspection of the algorithm's performance on hundreds of scans gave no cause for concern. However, we have not yet tested the algorithm against various CT protocols (especially low‐dose protocols) that may have an impact on the algorithm's ability to localize the spheres within the CT image volume.

Finally, we were limited by not being able to test our algorithm on a large set of FDG‐PET scans of a single phantom, nor were we able to test the algorithm against various PET reconstruction parameters. In particular, the FWHM postsmoothing parameter could potentially impact the algorithm, as evidenced by the decrease in positioning standard deviations between the 6 mm and 12 mm postsmoothing reconstructions. Another potential source of variability that was not examined is the ringing artifact that often occurs around the spheres when using reconstruction algorithms that include the scanner point spread function. We were limited by the available data (most of the scans used for testing this algorithm were actually performed for other projects and we performed our tests on the image volumes on hand). Even though these unexplored effects could potentially impact the performance of the algorithm, we believe the impact would be small, as seen in the 6 mm versus 12 mm smoothing case, where the variability remained under 0.5 mm for all PET scans.

Future work will include testing the algorithm under many reconstruction parameters on FDG‐filled phantoms. We will also explore the possibility of creating a comparable algorithm for use with the widely‐used American College of Radiology (ACR) phantom.

## V. CONCLUSIONS

We have proposed and demonstrated an algorithm that is robust, allows for repeatability of ROI measurements according to the NEMA/MITA NU‐2 standard for the image quality (IQ) phantom, and helps to eliminate variability due to positioning of the phantom within the scanner. The automated method also allows high throughput, batch processing, and minimal processing time by eliminating the need for user intervention in the drawing of the ROIs. For standard PET/CT image volumes, the algorithm takes between 3 and 4 min to run on a laptop computer with a 2.66 GHz processor and 4 GB of RAM.

For distribution, we have implemented the algorithm as a plug‐in to the Osirix DICOM viewing software package, available for download from http://depts.washington.edu/xcaliper.

The intent is that others will download, use, and test this plug‐in or implement the algorithm in other software. Widespread usage and testing of this plug‐in and algorithm will help the medical imaging community move toward a standard for NEMA IQ phantom measurements.

## ACKNOWLEDGMENTS

The authors would like to thank Tzu‐Cheng (Efren) Lee, Wendy McDougald, and Adam Alessio of the University of Washington for their help in the collection of the many PET and CT scans used in this work. This work was funded in part by NIH grants U01‐CA148131, R01‐CA169072, and SAIC Contract 24XS036‐004.
